# Nanoparticles for the treatment of glaucoma-associated neuroinflammation

**DOI:** 10.1186/s40662-022-00298-y

**Published:** 2022-07-02

**Authors:** Lidawani Lambuk, Nurfatihah Azlyna Ahmad Suhaimi, Muhammad Zulfiqah Sadikan, Azliana Jusnida Ahmad Jafri, Suhana Ahmad, Nurul Alimah Abdul Nasir, Vuk Uskoković, Ramlah Kadir, Rohimah Mohamud

**Affiliations:** 1 Department of Immunology, School of Medical Sciences, Universiti Sains Malaysia, Kubang Kerian, 16150 Kota Bharu, Kelantan Malaysia; 2 Centre for Neuroscience Research (NeuRon), Faculty of Medicine, Universiti Teknologi MARA, 47000 Sungai Buloh, Selangor Malaysia; 3 TardigradeNano LLC, 7 Park Vista, Irvine, CA 92604 USA

**Keywords:** Nanoparticles, Ocular drug delivery, Neuroinflammation, Glaucoma, Retinal ganglion cell

## Abstract

Recently, a considerable amount of literature has emerged around the theme of neuroinflammation linked to neurodegeneration. Glaucoma is a neurodegenerative disease characterized by visual impairment. Understanding the complex neuroinflammatory processes underlying retinal ganglion cell loss has the potential to improve conventional therapeutic approaches in glaucoma. Due to the presence of multiple barriers that a systemically administered drug has to cross to reach the intraocular space, ocular drug delivery has always been a challenge. Nowadays, studies are focused on improving the current therapies for glaucoma by utilizing nanoparticles as the modes of drug transport across the ocular anatomical and physiological barriers. This review offers some important insights on the therapeutic advancements made in this direction, focusing on the use of nanoparticles loaded with anti-inflammatory and neuroprotective agents in the treatment of glaucoma. The prospect of these novel therapies is discussed in relation to the current therapies to alleviate inflammation in glaucoma, which are being reviewed as well, along with the detailed molecular and cellular mechanisms governing the onset and the progression of the disease.

## Background

Glaucoma, a prime cause of irreversible blindness, refers to a group of ocular disorders with multifactorial etiology. As of now, it is considered a neurodegenerative disease in both the eye and brain [1]. In 2020, approximately 76 million people suffered from glaucoma and this number is expected to reach 112 million by 2040 [2]. These complex neurodegenerative disorders are characterized by optic neuropathy which is potentially progressive and visible changes can be seen at the optic nerve head (ONH) [3]. Glaucomatous optic neuropathy indicates a structural damage to the optic nerve with the corresponding loss of function. The structural damage is observed through the neurodegeneration of retinal ganglion cell (RGC) axons and deformation of lamina cribrosa with a concomitant diffuse and localized nerve fiber bundle pattern [4]. Undetected glaucoma in the early stages increases the risk of visual field loss [5]. The visual acuity may be spared at the early stage of the disorder, but progression of the neurodegenerative changes may result in the complete loss of vision [6].

**Fig. 1:**
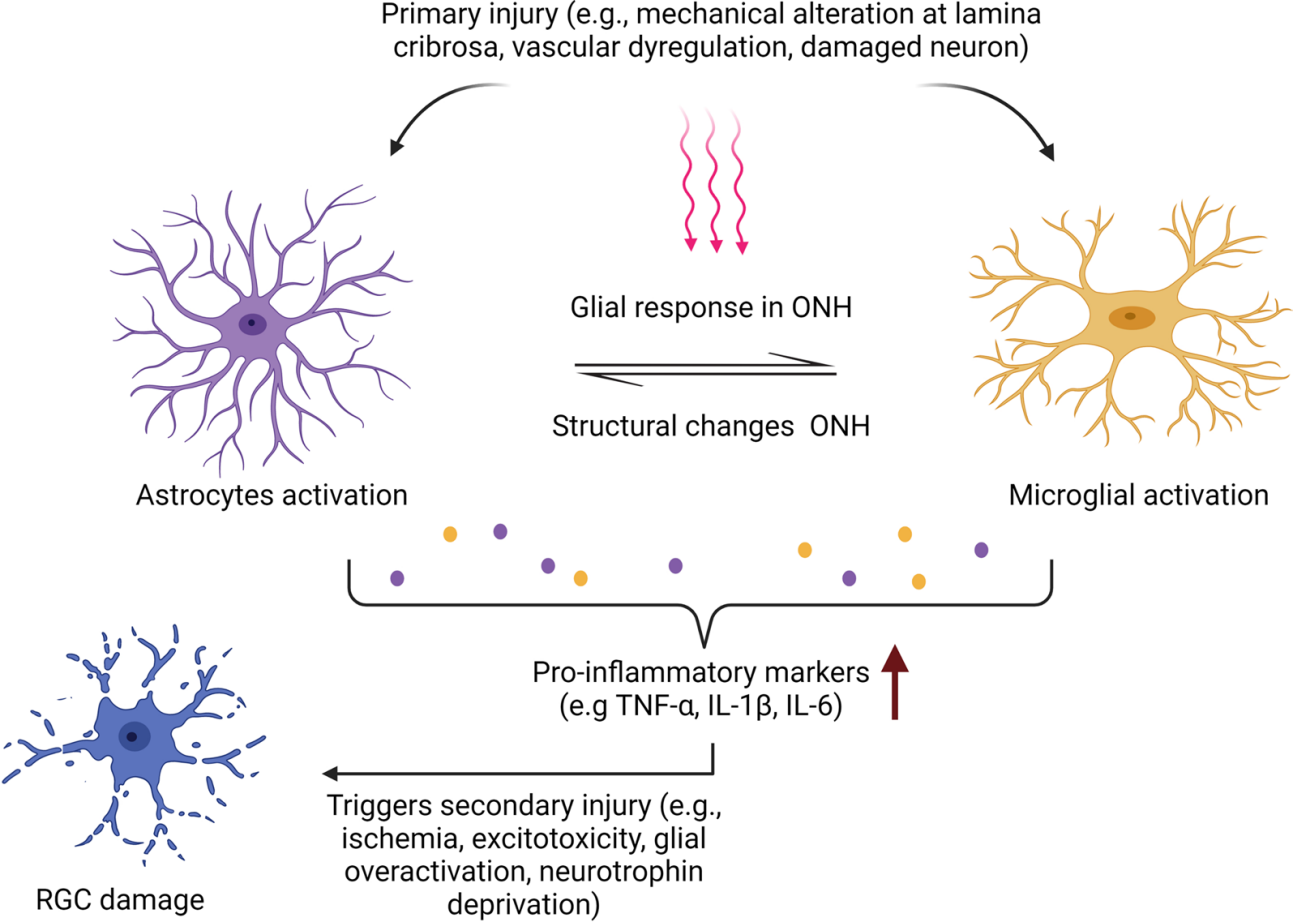
Relationship between microglia and astrocytes in glaucomatous neurodegeneration. Upon injury, they release immunological signals, which include pro-inflammatory cytokines, subsequently triggering secondary mechanisms exacerbating the neuronal injury and eventually cell death. Created with BioRender.com (2022)

Mechanisms underlying the development and progression of glaucoma at the level of RGC axons at the ONH remain unclear [7]. However, studies have indicated that early neuroinflammatory response is potentially a contributing factor to glaucomatous optic neuropathy (Fig. 1) [8]. Immunological surveillance in retina mediated by astrocytes, microglia, and other blood-derived immune cells is hypothesized to be associated with pro-inflammatory events leading to RGC damage [9, 10]. Several studies also found substantial evidence on the detrimental impact to axons, cell bodies, and dendrites of the ganglion cells during the early stage of experimental glaucoma in animal models [11, 12]. Interestingly, dampening certain pro-inflammatory pathways appears to have a neuroprotective effect on RGCs, particularly on events at the ONH during the early stages of glaucoma, further demonstrate the role of neuroinflammation in its pathogenesis [13]. Regardless of the initiation of insults, the neurodegeneration of ganglion cell axons is associated with the loss of ganglion cell bodies via apoptosis [14]. Since RGCs are unable to regenerate their axons, their loss is irreparable, which can disable the eye from generating connections to the brain and result in lifelong visual loss [14]. However, it calls for a greater concern because its prevalence is on the rise, and unlike in the case of cataracts, there is no effective therapy available [15].

The goal of any glaucoma treatment is to prevent vision loss. Most recent therapies have been focused on lowering intraocular pressure (IOP), as it is the only proven treatment for glaucoma; elevated IOP is considered the primary risk factor for the initiation and progression of the disease [3]. However, a significant number of glaucoma patients show worsening visual fields even when the IOP is controlled [16]. Although higher baseline IOP and older age are regarded as consistent and predisposing factors for glaucoma progression, several other factors should not be overlooked [17, 18]. In particular, individuals with family history of glaucoma, genetic predisposition, medications for pre-existing conditions such as systemic hypertension and diabetes, high myopia with great disc torsion of the optic disc and thinner lamina cribrosa at the ONH, and central corneal thickness, are amongst the factors reported that can influence the development of the disease [19, 20]. Nevertheless, to this day, IOP remains as the cardinal modifiable parameter in the management and treatment of glaucoma. In spite of that, most treatments for controlling IOP are associated with adverse effects and none of the current anti-glaucoma medications provide retinal neuroprotection by preventing RGC loss [21]. Although more than a few emerging therapeutic agents seem to have the potential to provide neuroprotection in human glaucoma, none of them have been clinically approved so far. Thus, there remains a need for therapeutic interventions that can provide maximal retinal neuroprotective effects in glaucoma with minimal adverse effects.

Glaucoma drug therapy typically employs topical instillations of eye drops. Although other glaucoma treatments such as surgical and laser therapy are increasingly utilized in the clinical setting, conventional eye drop remains the primary treatment for the majority of glaucoma cases [21]. Owing to various anatomical and physiological barriers in the eye, it is highly challenging for the drug to reach the target site [22]. Topical application of the glaucoma drug is predicted to reach the target tissue at the amount not higher than 5% of the applied amount due to the rapid clearance mechanisms at the corneal surface [23]. In addition, poor instillation by patients, especially the elderly, and the drug overspill are other contributing factors to the low bioavailability of ocular drugs in glaucoma [24]. To circumvent these obstacles, a new paradigm for glaucoma medical therapy is needed to fulfil the gold standard of the treatment criteria, including the efficient reduction of the IOP in such a way that the visual field is not compromised, and the optic nerve protected without causing tachyphylaxis and without generating other local and systemic adverse effects. It is also important to consider a treatment that can promote patient compliance and applicability in diverse patient populations [25].

Among many new therapeutic innovations for the treatment of glaucoma, nanoparticles (NPs) occupy a prominent place [26]. In this review, we have examined the potential of NPs in the treatment of glaucoma by emphasizing the mitigation of neuroinflammation, all to circumvent the drawbacks in the current glaucoma therapies.

## Main text

### Modulation of neuroinflammation in glaucoma

**Fig. 2:**
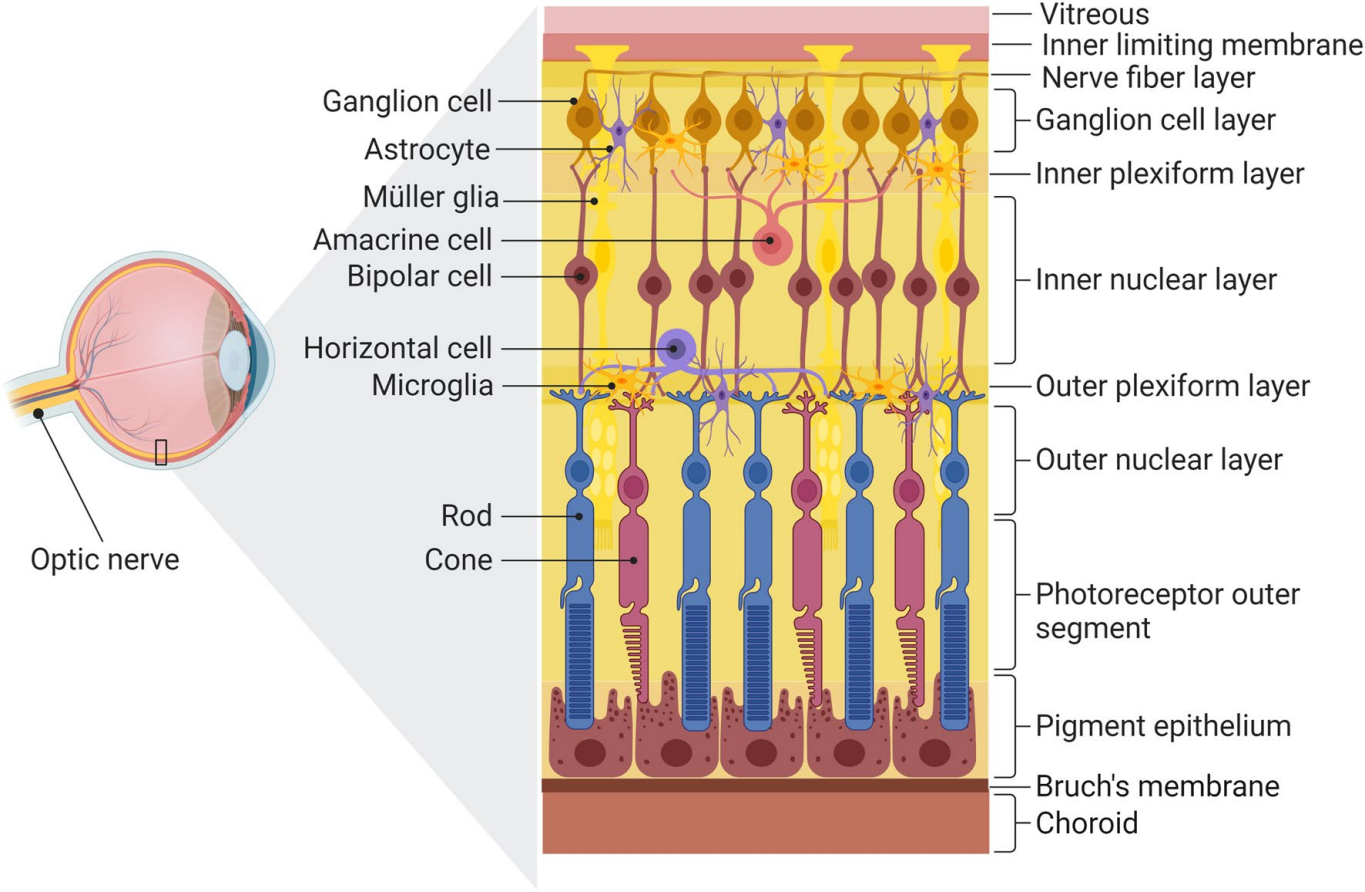
The overview of human retinal cells and layers (Adapted from “Structure of The Retina”, by BioRender.com (2022). Retrieved and edited from https://app.biorender.com/biorender-templates)

Neuroinflammation in glaucoma can take place at different physiological locations, but it is most prominent at the posterior segment of the eye (i.e., retina and optic nerve; Fig. 2) and the brain (i.e., superior colliculus and lateral geniculate). It can also occur peripherally in blood vessels. Nonetheless, the primary focus has been on the RGCs. In the ONH, most research has demonstrated the critical concern of glaucoma in which the RGC soma, synapses and dendrites show the effects of neuroinflammation and peripheral immune responses. Recent studies have demonstrated leukocytic recruitment into the ONH and the retina, which may contribute to the development of the glaucoma [13, 27].

In the pathophysiology of glaucoma, RGC axons are the first to be affected. Mechanical alterations to lamina cribrosa, neurotrophic signaling, direct RGC pressure, and neuroglial activation, such as that of microglia or astrocytes, are among the initial stimuli for this event. Müller glia, astrocytes, and microglia are the ‘resident cells’ that stimulate innate immune responses in the retina and optic nerve. The astrocytes play a crucial role in controlling homeostatic conditions for neurons by maintaining neurovascular coupling (neuronal activity/local blood flow) [28, 29]. When activated, astrocytes undergo morphological alteration and proliferation to the area of injury. Severe astrocytosis, an abnormal increase in the number of astrocytes that leads to inflammatory responses, has been reported in glaucoma and is known to be involved in the onset of the disease [30]. Some studies have reported that ONH astrocytes have a phagocytotic effect which are able to engulf synaptic materials and cellular debris [31, 32]. Nevertheless, the extent of effects of astrocytosis is still the subject of ongoing research.

In glaucomatous pathophysiology, microglia and macroglia are the key players responsible for immunoregulation in the retina [33]. These cells are responsible in several key functions including providing nutritional and structural support, regulating metabolic activity and homeostasis, phagocytosis as well as levels of cytokines and neurotrophic factors [34].

### Major neuroinflammatory cells in glaucoma

Microglia is the key cell type controlling neuronal function and homeostasis. They are phagocytes that play a vital role in the innate immune response. As resident macrophages, its presence is undoubtedly ubiquitous in the central nervous system (CNS) [35]. This cell is the first to react toward the site of injury by stimulating inflammatory cascades and recruiting other inflammatory cells, such as astrocytes. In an in vivo study of glaucoma, activated microglia were found to increase in number in glaucoma; however, it is not certain whether these reactions are beneficial [36]. Initially, microglia were assumed to have ascended from the yolk sac of macrophages that had entered the brain during the development of fetus. However, more recently, it is believed to have come from circulated monocytes, which later differentiated into microglia [37]. Microglia are responsible for homeostasis of the neural circuits and angiogenesis in the retinal development. In mature retina, microglia help in neuronal signaling and integrity of synaptic transmission [38–40]. Some studies have suggested that inactivation of retinal and ONH microglia using drugs known as minocycline lowers the neurodegenerative actions [41, 42]. Astrocytes, microglia, and macrophages are believed to be involved in neuronal inflammation, with aging among the causative factors [43].

In glaucomatous eyes, activation of microglia has been detected at an earlier stage, whereas the aggregation, activation and redistribution of microglia is seen even before RGC injury has taken place in a DBA/2J mouse model of chronic hereditary glaucoma [44]. The early phase of the glaucomatous model in mice also involved the monocytic recruitment and other pathologies with neuronal damage [45]. Microglial activation and proliferation can lead to detrimental effects on RGCs through the secretion of pro-inflammatory cytokines, such as interleukin-6 (IL-6), tumor necrosis factor-alpha (TNF-α) and reactive oxygen species (ROS) [46]. In another study utilizing the same animal model, the transcriptome of ONH microglia was seen to change drastically in the metabolic, phagocytotic, inflammatory and sensome pathways [47]. This was confirmed by another study, which showed an increased activity of microglia and their density in the retina, including the optic nerve when IOP was elevated [36]. This finding supports the hypothesis that the chronic ocular hypertension inhibits the homeostasis-regulating function of microglia [47]. Despite all these findings, the role of microglia is still debatable. Few studies have suggested that RGC injury can be worsened by microglia when the inflammatory mediators such as TNF-α, interleukin 1β (IL-1β), IL-6, matrix metalloproteinases, Fas ligands (FasL), and ROS are released [46, 48].

Macroglia are predominantly Müller cells and astrocytes that share similar transcriptomic profiles and functions [49]. Out of these two cell types, the prime macroglial cells are Müller cells, which can be found across the retina. Their cell bodies lie in the inner nuclear layer, which elongates into two trunks that extend their ends into the inner limiting membrane. The inner limiting membrane separates the retina from the vitreous body and is one of the most significant barriers for ocular drug delivery [50]. For a detailed overview of the inner limiting membrane, the reader is referred to the work by Peynshaert et al. [50–52]. Müller cells are essential in maintaining the structural integrity of the retina. They are also important regulators for cell metabolism in the retina [53]. Müller cells are anatomical conduits among the retinal neurons, including the cellular environment. Hence, they help in maintaining retinal homeostasis. Astrocytes are the foremost glial cells found at the ONH. As the dominant component of glial cells in the CNS, astrocytes engage in a variety of critical functions, such as ionic balance regulation, metabolic supply and its structural maintenance, neurotransmitter transmission, and synaptic plasticity [54]. Collectively and together with microglia, astrocytes and Müller cells ensure a smooth process for the synaptic activity to occur by maintaining ion and neurotransmitter levels.

When an injury occurs to the retina, macroglia can be stimulated to release glial fibrillary acidic protein (GFAP) and other extracellular matrix proteins [55, 56]. In the glaucomatous retina, there is an increased amount of GFAP immunostaining and macroglia display a hypertrophic morphology, suggesting the existence of retinal gliosis in glaucoma [57]. Several models of glaucoma have showed that the number of astrocytes increases with increased GFAP immunoreactivity, and this was also seen on the extracellular matrix remodeling on the ONH. GFAP is an intermediate filament of the glial cell cytoskeleton that upsurges when astrocytes transform into their reactive state [55]. Furthermore, some studies have shown that ONH astrocytes play a significant role in the engulfment of optic axons, including stimulation of axon degradation, which is one of the proposed mechanisms for the sectorial nature of RGC loss in glaucoma [32]. The inflammatory response in glaucoma is activated and mediated early on through a process called astrogliosis. Several inflammatory pathways were activated by astrocytes when rats were injected with hypertonic saline into their episcleral veins, leading to a high IOP [58]. The activation of inflammatory pathways includes tumor necrosis factor α (TNF-α) signaling, nuclear factor kappa B (NF-κB) activation, autophagy, and inflammasome-associated regulators.

### Inflammatory pathways

TNF-α and toll-like receptors (TLRs) pathways are among the crucial complement cascades in glaucomatous neuroinflammation. Furthermore, there are various associated inflammatory mediators involved such as cytokines and prostaglandins [59], and pathways such as β2-microglobulin and cluster of differentiation 3 (CD3) [60]. Studies are currently focused on the classical pathway of the complement cascade whereby RGCs detect stimuli of injuries and activates the complement component 1 (C1) complex, a giant proteolytic enzyme [61–63]. This is followed by activation of the C3 convertase, which can attract leukocytes and further activates C5 convertase. C5 convertase would recruit more leukocytes and stimulate cell lysis through the membrane attack complex. In the complement cascade in a glaucomatous eye, glial cells such as astrocytes would amplify the RGC signal to boost microglia response and even attract monocytes, especially in the ONH and inner plexiform layer. Rather than killing the RGCs, the complement system is believed to protect them from further damage and maintain their function [64]. Still, based on the previous glaucomatous animal model, the nature of the neuroinflammatory mechanism is thought to possess an evidently damaging role on the disease [65, 66].

Meanwhile, TLRs pathway induce glaucomatous neuroinflammation in two ways, either through polymorphism of TLR4 alleles [67] or increase in TLR4 protein expression in the retina of glaucoma animal models [68]. Different TLRs recognize different stimuli. For instance, TLR3 detects double-stranded RNA of foreign substances, whereas TLR4 focused on detecting an endogenous ligand, for example, tenascin-C, which is elevated in the glaucomatous ONH [69, 70]. It has also been found that monocytes and microglia have more detecting TLRs as compared to astrocytes. Even though some studies have examined TLRs in RGC injury, more investigation is needed to further delineate the role of TLRs in human glaucoma.

Another critical activator of neuroinflammation in glaucoma is TNF-α, which is produced by astrocytes and especially microglia [71, 72]. Studies have reported that polymorphism [73, 74] and increment of TNF-α in the vitreous body, retina and optic nerve are associated with glaucoma [75, 76]. Parallel to FasL’s downstream actions, TNF-α also triggers RGC cell death [77, 78]. In a vitreous glaucoma mouse model, oligodendrocyte and RGC damages are induced by soluble murine TNF-α while TNF-α suppression prevented these damages [43, 79]. This is similar to the extent of blocking FasL activity by pharmacotherapy [80]. Although TNF-α inhibitors seem to have a neuroprotective property in clinical settings, further research is needed to clarify this, especially in glaucomatous diseases [74].

Several pathways of neuroinflammation have been proposed, although more extensive studies are required for both in vivo and human glaucoma. The continued elucidation of these pathways is essential for defining therapeutic targets that have clinical benefit.

## Current investigational therapies to alleviate inflammation in glaucoma

### Immunomodulatory drugs

With respect to the neurodegenerative potential of neuroinflammation, several molecules have showed the potential to act as ‘neuroprotectors’. Citicoline (cytidine 5′-diphosphocholine) exemplifies a naturally endogenous compound that has been evaluated for its protective role on RGC in glaucoma [81]. A number of in vitro and in vivo studies have demonstrated the neuroprotective role of citicoline via increased dopamine retinal levels, enhanced anti-apoptotic effect, restrained thinning of the retinal nerve fiber layer (RNFL), regeneration of neurites, defense against glutamate excitotoxicity, and minimized RGC impairment, thereby enhancing a better visual field [82]. A significant reduction in the apoptotic nuclei pathway of cell death with contrasting synaptic loss achieved with citicoline treatment showed its efficacy in protecting against the excitotoxic neuronal damage and thus delayed the progression of glaucoma [83, 84]. Citicoline also plays a crucial role in the regeneration of the axon through sphingomyelin synthesis, which stabilizes the plasma membrane of RCG axons, thereby suppressing free fatty acids and protecting against the redox imbalance [85]. Experimental studies in adult male Albino rabbits treated with citicoline had demonstrated a higher dopaminergic neurotransmission in the brain compared to the untreated group, and highlighted the influence of citicoline on retinal catecholamine levels [86]. The usage of citicoline against retinal damage eventually proved to have a neuroprotective effect in kainic acid-induced neurotoxicity in vivo [87]. Shuettauf et al*.* investigated the anti-apoptotic effect of mitochondria-dependent cell death mechanism by delivering citicoline with lithium, with the outcome being a rise in the RGC density [88]. In a randomized clinical trial, Parisi et al*.* observed, enhanced retinal and visual functions in a glaucoma patient who received citicoline [89]. A follow-up electrophysiological analysis of glaucomatous visual dysfunction, which was carried out in conjunction with hypotensive therapy, further confirmed citicoline to be a fitting medical treatment for glaucoma within an extended period of time [90]. The effects of citicoline administered through oral and intramuscular approaches were subsequently tested on glaucoma patients with moderate visual defects, showing improvement in retinal function [91]. In a similar study conducted by Ottobelli et al. patients with progressing glaucoma were supplemented with an oral citicoline solution and the follow-up visual examinations showed reduced rate of mean progression by the end of the study after the treatment [92]. Lanza et al. demonstrated neuroprotective effect of oral citicoline, which slowed down the progression of primary open-angle glaucoma (POAG). The citicoline therapy assessment by standard automated white-on-white perimetry showed a stable and highly significant mean deviation (MD) of progression over time in treated patients compared with untreated patients [93]. Another method carried out in a different study acknowledged that intravenous therapy can be a means for citicoline to reduce the progression of glaucoma in conjunction with citicoline eye drops as the IOP lowering treatment. Visual field and RNFL loss detected were much lower on average [94]. Studies involving the encapsulation of citicoline eye drops in a liposomal formulation conducted by Parisi et al. also suggested improved retinal bioelectrical responses with enhanced visual cortex bioelectricity [95]. Overall, these findings showed the crucial role of citicoline as a neuroprotective compound for managing glaucoma, yet further clinical trials with larger sample sizes are highly needed to gather more understanding in relation to the dose-response and clinical effects.

The renin-angiotensin aldosterone system (RAAS) is a complex endocrine system that has a major function in the regulation of hemodynamic stability and fluid balancing. Upon the occurrence of hypotension in the body, granular cells of renal juxtaglomerular apparatus release the renin enzyme, which cleaves angiotensinogen to angiotensin II (Ang II) via Ang II type 1 receptor (AT1-R) [96]. Recent evidence suggests the prospect of utilization of AT1-R antagonists as a treatment for several conditions such as hypertension, blood pressure and cardiovascular diseases, mainly due to the pro-inflammatory effects of Ang II and aldosterone [97]. Studies have proven that administration of AT1-R blockers is not only able to transverse the blood-brain barrier and communicate with AT1-R to minimize the infarct volume, but also extenuate inflammatory and oxidative stress in the retina and brain [98]. Yang et al. showed that the AT1-R signaling blockade of candesartan succeeded in averting the retinal neuronal death in a rat model of chronic glaucoma [99]. Similarly, the orally active AT1-R antagonist candesartan inhibited toll-like receptor 4 (TLR4-apoptosis signal-regulating kinase 1 pathway), which supported the activation of RAAS in the innate immune response, expediting neural cell death [96]. The conclusion is a significant neuroprotective effect of Ang II against RGC loss.

### Natural products

Aside from the standard therapy used currently, which involves IOP reduction through medical drugs, laser and surgical therapy, herbal medicine is one of the primary alternatives chosen in the management of glaucoma [100]. In the nineteenth century, active compounds were directly isolated from plants [101]. Plants such as ginkgo biloba, saffron, and phytochemicals such as epigallocatechin-3-gallate and resveratrol are known as traditional remedies used in glaucoma pathology [102].

Among various antioxidative compounds present, Ginkgo (Ginkgo biloba), which originated from China 250 million years ago, has been recognized for its therapeutic effects in several pathologies, including neurodegenerative diseases [103]. The beneficial component of this living fossil tree is found in the ginkgo extract, which contains polyphenolic flavonoids that stabilize the mitochondria at organelle level, and also exerts multiple therapeutic properties, including the antioxidant, antimicrobial, neuroprotective and antiapoptotic effects [104, 105]. Extract 761 (EGb761), obtained from leaves of the ginkgo plant, has been effective in treating Alzheimer’s dementia and cognitive impairment. Therefore, researchers attempted to use EGb761 in the treatment of glaucoma due to the analogous biological and mechanistic features between these two chronic disorders [106]. Namely, both Alzheimer’s dementia and glaucoma are age-related pathologies, experiencing the RGC degeneration and deposition of extracellular fibrils in the exfoliation syndrome, indicating that both are likely derived from similar misfolding mechanisms [107]. In previous studies, both short- and long-term effects of the ginkgo biloba extract (GBE) were tested and the extract was used to treat pre-existing patients with normal tension glaucoma (NTG), often resulting in a significant improvement of visual acuity [108, 109]. However, Guo et al. who performed a randomized, crossover clinical trial, failed to demonstrate the effect of GBE to improve progressing visual defects within normal NTG patients, likely due to the smaller sample size and shorter time periods applied during the study [110]. The administration of GBE also showed an increasing end diastolic velocity in the ophthalmic artery and NTG throughout clinical cross-over trials, highlighting the desirable effect of the drug on the retinal blood flow in glaucoma disorders [111, 112]. Shim et al. supported these findings by showing the escalating MD upon GBE and bilberry anthocyanin treatment [113]. In a similar study, the standardized EGb761 extract demonstrated a progressing pharmacological effect on the oxidative stress with improved vascular circulation in both in vitro and in vivo experiments, highlighting the neuroprotective effect of the drug against the hypoxic injury of RGCs [114]. These findings have emphasized the prospect of this natural medicine in treating glaucoma. However, its usage has yet to become widely recognized in public.

Saffron, the dried stigmas originating from the *Crocus sativus* flower of the Iridaceae family in Greece, has been commonly used in cooking as an aromatizing and coloring seasoning [115]. The major constituents in saffron are natural carotenoid compounds, namely crocin and crocetin [116]. Its usage in the medical field has been recognized in the treatment of various diseases due to the wide therapeutic spectrum, including neuroprotective, anti-inflammatory, anti-oxidant and anti-genotoxic activity [117]. Both saffron compound extracts, crocin and crocetin, showed an enhanced neuroprotective effect through repression of activated microglia neurotoxicity. The development of intracellular ROS and nitric oxide is inhibited with a slower release of TNF-α and IL-1β [118]. These beneficial aspects can be observed in animal models of neurodegenerative ocular diseases and patients suffering from diabetic retinopathy and age-related macular degeneration (AMD). Studies in animal models with retinal damage emphasized the role of crocin in saffron as an inhibitor of the ischemic damage and a stabilizer of the ocular blood flow, alongside the neuroprotective effect provided by crocetin [119]. In a pilot study, Bonyadi et al*.* investigated the influence of an aqueous saffron extract on the IOP in the eyes of POAG patients and showed that the treatment significantly decreased the mean baseline IOP compared to the control group by the end of the therapy [120]. Despite the limited studies on saffron in glaucoma disease, the saffron extract emerges as an important therapeutic agent for potential clinical use.

Epigallocatechin-gallate (EGCG) is a type of catechin mainly found in green tea. It is well known as a robust antioxidant with multifunctional properties and has been investigated for its contribution to neuroprotection in human corneal epithelial cell culture models and animal models of glaucoma [121, 122]. Earlier findings not only demonstrated its therapeutic effect on the axon and the bodies of RGCs in optic nerve crush and *N*-methyl-d-aspartate (NMDA) toxicity studies, but also showed an elevation in the survival rates of RGCs via oral administration [123, 124]. In a similar study, which also used oral EGCG, the drug was shown to be a potent penetrator into the retina, where it reduced both the injury caused by ischemia and in vitro white light-induced apoptosis in RGC-5 cells [125]. Falsini et al. claimed a higher amplitude detection in the OAG group compared to the ocular hypertension (OHT) group in a pattern electroretinogram analysis, thus supporting the prospect of short-term supplementation of EGCG [121]. ECGC does not only provide protection against the oxidative stress, but also has the capability to weaken the glutamate-induced cytotoxicity by decreasing the ionotropic calcium influx [126]. These outcomes showed that EGCG is a suitable neuroprotective agent for the glaucoma treatment. However, there is a need to perform further studies to determine the long-term benefits, the component activity, and the precise dosage requirements for EGCG in the glaucoma treatment.

Resveratrol (RSV), also known as 3,5,4′-trihydrocystilbene, a nonflavonoid polyphenol compound derived from plant sources such as grapes, blueberries and apples, has been developed into an effective phytoalexin [127]. It has diverse roles in relation to the well-being of humans, by virtue of biological attributes including antioxidant, anti-inflammatory and neuroprotective functions [128]. In POAG patients, RSV was shown to interrupt intracellular ROS, inhibit the release of inflammatory cytokines and slow down the accretion of carbonylated proteins, hence supporting the neuroprotective action of the drug against the RGC apoptosis and the ability to slow down the progression of glaucoma [129]. Other studies also demonstrated this neuroprotective effect of RSV, including the delay in the RGC loss upon dosing with RSV and riluzole. Although both single and combined administrations were effective, an improved and better RGC protection was provided through the combined therapy [130]. Luo et al*.* showed that sirtuin 1 (SIRT1) activation by RSV confers neuroprotection in mice with ischemia–reperfusion injury (IR) via Akt activation and mitochondrial apoptotic suppression with a verified concentration of intravitreal injection, and thus contributed to the understanding of the mechanism of action important for the clinical usage of RSV [131]. Inhibition of endothelin-1, a vasoactive peptide in glaucoma, highlighted a pivotal effect of RSV [132]. Moreover, researchers have suggested the induction of mitochondrial biogenesis by RSV to alleviate glaucomatous retinopathy. This is due to the efficiency of RSV in reducing derivative-serum in the RGC-5 cell line by subcellular translocation of SIRT1 dependent proliferator-activated receptor-gamma coactivator 1 alpha [133]. In addition, Shamsher et al. studied the in vitro and in vivo neuroprotective effects of RSV and curcumin nanoparticle formulations with ~ 70% encapsulation efficiency [134].

One of the examples of long-standing, well-conducted research and development of an anti-inflammatory agent comes from curcumin, a major active compound of turmeric, Curcuma longa [135]. Curcumin has shown exceptional promise for the beneficial modulation of numerous signaling molecules (e.g., pro-inflammatory cytokines, NF-κB, apoptotic proteins, and C-reactive protein) in multiple diseases, including cancers and inflammatory and neurodegenerative disorders. Apart from anti-inflammatory properties, curcumin exerts antioxidant, anti-microbial and anti-tumorigenic activity. Owing to these properties, curcumin has been extensively studied in vitro and in vivo in the context of many inflammatory, autoimmune, and degenerative diseases of both anterior and posterior segment, and has been suggested as an adjuvant therapy [136]. In a retinal ischemic injury animal model, curcumin was reported to prevent ischemic damage to the RGC and microvasculature via suppression of NF-κB signal transducer as well as activation of transcription 3, and monocyte chemotactic protein 1 expression [137]. The chemical properties in curcumin with anti-inflammatory and antioxidant functions have been suggested to be associated to its hydroxyl and methoxy group, which deregulates TNF-α and pro-inflammatory interleukins which lead to the downregulation of STAT pathways. In both in vitro and in vivo experimental glaucoma studies, curcumin has shown antioxidant effects, as demonstrated by the improved cell viability of microglial cells, reduced intracellular ROS and apoptosis of RGCs [138]. These findings should make an important contribution to the therapeutic potential of curcumin in clinical ophthalmology, notwithstanding that this potential is restricted by a few adverse factors, including extremely poor bioavailability and water solubility [139]. The active fraction of curcumin detected in the blood is often suboptimal, for which reason increased doses are needed to achieve the proper therapeutic effect [140]. To overcome these limitations, several approaches such as the use of enhancers, analogues and nanocarriers to provide a hydrophobic environment for poorly water-soluble curcumin have been reported and extensively reviewed by other [141–143]. An in vivo study by Davis et al*.* demonstrated < 95% encapsulation efficiency with good stability upon the formulation of topical curcumin-loaded, Pluronic-F127 stabilized d-α-tocopherol polyethene glycol 1000 succinate NPs (< 20 nm) [143]. Similarly, Cheng et al*.* developed a formulation consisting curcumin-latanoprost NPs (~ 161 nm), which resulted in a sustained-release profile with low oxidative stress-mediated damage via ROS production and apoptosis in vitro and in vivo [144]. These studies highlight the potential of curcumin to provide a neuroprotective therapy in glaucoma. Of note, the use of nanocarriers is one of the most prospective approaches in improving curcumin delivery. With the development of a nanocarrier suitable for utilization as a topical formulation, the bioavailability of curcumin could be drastically improved [143]. One of the promising drug carriers for the delivery of curcumin has been the amphiphilic polymer polyvinyl caprolactam-polyvinyl acetate-polyethylene glycol graft copolymer, Soluplus [145]. Although this and other types of carriers must be further investigated, they provide important opportunities for advancing the understanding of curcumin as an anti-inflammatory agent and, potentially, as a neuroprotective therapy in glaucoma.

The above mentioned substances were extensively studied in both IOP-dependent and -independent types of glaucoma. Outside traditional clinical settings, the progression of glaucoma can be controlled, yet it still cannot substitute the conventional therapeutic management of glaucoma; hence, further studies are required. It is noteworthy that an increasing number of experimental studies currently consider molecular targets in the modulation of inflammatory responses activated by microglial cells, RGCs and other retinal cells that elicit downstream actions of inflammatory pathways responsible for glaucomatous neurodegeneration (Table 1). Although the therapeutic anti-inflammatory potential of the various agents seems to be encouraging, their neuroprotective effects could be attributed to other factors, including the route of delivery to the target tissues, which may have an impact on patient safety and compliance in clinical practice [3]. Most of the therapeutic agents also require a proper formulation to provide optimal neuroprotection, particularly in promoting RGC survival under glaucomatous conditions. To fully explore the potential of these therapies and their biocompatibility for the treatment of glaucoma in humans, further investigations are necessary to develop formulations that can be administered non-invasively.

**Table 1:** Recent therapeutic options on anti-inflammatory and neuroprotective effects in experimental models of glaucoma and other ocular disease-associated RGC loss

Therapeutic agent	Experimental model	Route of delivery	Anti-inflammatory and neuroprotective effects	Refs.
Magnesium acetyltaurate (MgAT)	Retinal ischemia injury; retinal excitotoxicity injury in rat	Intravitreal injection	• Suppressed ET-1- and NMDA-induced retinal and optic nerve damage through induction of iNOS, suppression of NF-κB p65, p53, AP-1 (c-Jun/c-Fos) signaling pathways, downregulation of TNF-α, IL-1β, IL-6, and caspase-3 • Preserved RGC survival by ~ tenfold in NMDA-induced group • Improved visual function after 7 or 14 days of treatment	[217, 218]
Dietary supplementation (combination of forskolin, homotaurine, spearmint, and B vitamins)	IOP elevation in mice	Oral	• Maintained IOP at baseline level 2 weeks before and after supplementation • Suppressed elevated IOP-induced NF-κB signaling pathway and reduced caspase-3 activity • Preserved retinal function and 20% RGC survival more than the untreated group	[219]
Laquinimod (LQ)	Retinal ischemia and reperfusion injury in mice	Topical	• Reduced numbers of activated microglia • Suppressed retinal TNF-α, IL-1β, IL-6, and iNOS levels • Inhibited caspase 8 and NLRP3 in retinae and microglia • Promoted RGC survival ~ 1.9-fold and preserved retinal function	[220]
ONL1204 (small peptide Fas antagonist)	IOP elevation in mice	Intravitreal injection	• Abrogated microglial activation by ~ 1.9-fold • Downregulated cytokines and chemokines, macrophage inflammatory protein (MIP), MIP-1α, MIP-1β, MIP-2, monocyte chemoattractant protein-1 (MCP1), interferon gamma-induced protein 10 (IP10), TNF-α, IL-1β, IL-6, and IL-18), caspase-8, components of the complement cascade (C3 and C1Q), TLR4, and NLRP3 • Prevented axon degeneration (*P* < 0.0001) and preserved RGC survival (*P* < 0.001) • No significant different in IOP	[221]
Apolipoprotein E (ApoE)-mimic peptide COG1410	Optic nerve crush injury in mice	Intravenous injection	• Reduced JNK phosphorylation, TNF-α, IL-1β, IL-6, iNOS, and Bax/Bcl-2 ratio • Promoted RGC survival by ~ 61% and reduced optic nerve damage (*P* < 0.05) • Preserved visual function	[222]
Caffeic acid phenethyl ester	Optic nerve crush injury in rat	Intraperitoneal injection	• Downregulated retinal glia-mediated NF-κB activation, IL-8, IL-6, iNOS, COX-2, and TNF-α • Attenuated gliosis (*P* < 0.01) • Enhanced RGC survival (*P* < 0.001)	[223]
Green tea extract (Theaphenon E)	Retinal ischemia and reperfusion injury in rat	Intragastric administration	• Downregulated TLR4, TNF-α, and IL-1β levels • Reduced expression of cleaved Caspase-3 and Caspase-8 • Downregulated expression Superoxide dismutase 2 (SOD-2), Janus kinase (Jak) and p38 • Enhanced RGC survival (*P* < 0.001) in ischemic retina	[224]
Kaempferol	Retinal ischemia and reperfusion injury in mice	Intragastric administration	• Downregulated expression levels of TLR4, TNF-α, IL-1β and IL-6 • Inhibited activation of NF-kB and JNK signaling pathways • Reduced active caspase-3 and caspase-8 • Prevented NLRP1/NLRP3 inflammasome activity • Prevented IOP-induced RGC death (*P* < 0.01)	[225]
Minocycline	Retinal vein occlusion in rat; retinal ischemia–reperfusion injury in mice	Intravenous injection	• Reduced activation of microglia • Reduced RGC loss (~ 45%, *P* < 0.05) • Improved visual function	[226]
Omega-3 polyunsaturated fatty acids	Anterior ischemic optic nerve injury in rat	Oral gavage	• Downregulated TNF-α, IL-1β, and iNOS levels • Reduced macrophage polarization • Survival of RGC in central and midperipheral retinas was ~ 2.3—(*P* = 0.03) and 2.0-fold (*P* = 0.03) higher • Reduced postinfarct apoptosis of RGCs by ~ 2.9-fold (*P* = 0.007)	[227]
Synthetic sterol (HE3286)	IOP elevation in rat	Oral gavage	• Maintained IOP at baseline level (*P* = 0.997) after oral delivery • Increased brain-derived neurotrophic factor (BDNF) expression and reduced TNF-α expression in the ONH • Reduced retinal IL-6, IL-1β, and p75 expression levels • Reduced microglia activation and reduced NF-kB localization • Increased NF-kB localization to neuronal nuclei in the superior colliculus and retina	[228]
4-(Phenylsulfanyl)butan-2-one	Optic nerve crush in rat	Subcutaneous injection	• Inhibited iNOS/COX-2 pathway in microglia • Increased RGC survival by ~ 36% in the central retina and ~ 35% in the mid-peripheral retina • Reduced RGC apoptosis by ~ 2.2-fold • Preserved visual function • No data on IOP comparison	[229]
Caffeine	Ocular hypertension in rat	Oral	• Partially reduced IOP level ~ 1.3-fold (*P* < 0.001) • Inhibited OHT-induced microglial activation • Reduced retinal TNF-α, IL-1β, and iNOS expression levels • Preserved RGC loss by ~ 1.8-fold (*P* < 0.05) but not RGC retrograde transport	[65]
Granulocyte colony-stimulating factor (G-CSF)	Optic nerve crush injury in rat	Subcutaneous injection	• Suppressed microglia activity • Downregulated TNF-α, IL-1β and iNOS expressions • Protected RGC from secondary degeneration injury by ~ 38% (*P* < 0.01) • No data on IOP comparison	[230]

*AP-1* = activator protein 1; *Bax* = bcl-2 associated x; *Bcl-2* = b-cell lymphoma-2; *C1* = complement component 1; *C1Q* = complement component 1Q; *COX-2* = cyclooxygenase-2; *ET-1* = endothelin-1; *IOP* = intraocular pressure; *IL* = interleukin; *iNOS* = inducible nitric oxide synthase; *JNK* = c-Jun N-terminal kinase; *NF-κB* = nuclear factor kappa B; *NLRP* = NOD-, LRR-family pyrin domain; *NMDA* = *N*-methyl-d-aspartate; *MCP* = monocyte chemotactic protein; *MIP* = macrophage inflammatory protein; *RGC* = retinal ganglion cell; *TNF* = tumor necrosis factor; *TLR* = Toll-like receptor; *OHT* = ocular hypertension; *ONH* = optic nerve head

## Potential of nanoparticles in drug delivery

### Current perspective on glaucoma therapies targeting neuroprotective agents

Neuroprotection in glaucoma refers to any IOP-independent intervention that preserves the optic nerve by preventing or delaying RGC and axonal degeneration [146]. Regardless of the various definitions, neuroprotection is a therapeutic approach directed at keeping RGCs alive and functional in progressive glaucomatous optic neuropathy [147]. Data from randomized controlled clinical trials show that even with excellent IOP control the disease is still exacerbated in some patients [148]. Therefore, the idea of IOP-independent treatment strategies in glaucoma should be extensively investigated.

There is a vast amount of literature on identifying neuroprotective agents targeting the mechanisms proposed that underlie RGC damage in glaucoma [149]. Glutamate excitotoxicity antagonists, neurotrophic factors, and oxidative stress suppression are some of the studied neuroprotective agents with favorable neuroprotective activities. For further details regarding these, the reader is referred to the comprehensive reviews by Sharif and others [3, 150–153]. However, results from human clinical trials have been inconclusive and non-consequential [154]. Over time, literature reports have shifted towards inflammatory and immune responses, supporting the notion that neuroinflammation could be the key player in the mechanism underlying retinal damage in glaucoma, potentially having a reciprocal causative role in the pathology [69]. Since the mediators of neuroinflammation activate the immune system within the CNS, they may have either harmful or beneficial effects on RGC survival. This justifies a dire need for better therapeutic strategies. As such, the development of new therapies aimed at modulating rather than suppressing neuroinflammation might also produce the highly sought-after neuroprotective effects.

### Challenges in ocular drug delivery

**Fig. 3:**
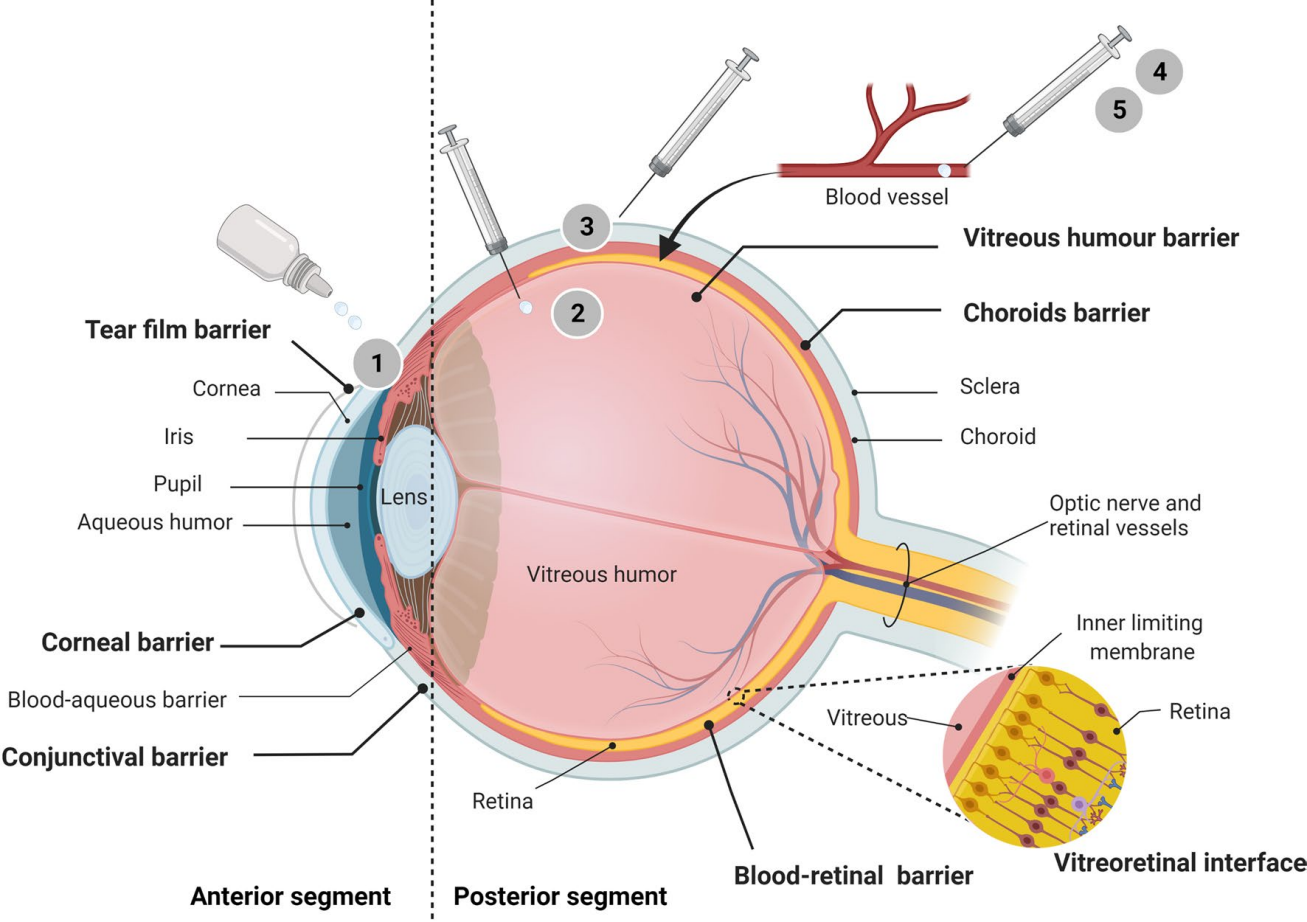
Schematic diagram of the human eye with barriers [tear film (precorneal), corneal, conjunctival, blood aqueous, vitreoretinal interface to blood-retinal barrier] and the common route of administration. Primary methods of drug delivery to the eye are topical (1), local ocular [e.g., intravitreal (2) and subconjunctival (3)] and systemic [i.e., oral (4) and intravenous (5)]. Topical instillation is the most widely preferred non-invasive route of drug administration to treat diseases affecting the anterior segment and, potentially, the posterior segment. The ocular barriers block the entry of the most active molecules; hence, effective drug delivery systems are required to facilitate the passage of the drug across these barriers and transport the given pharmaceutical compound to its target site to achieve an optimal therapeutic effect [151] (Adapted from “Anatomy of the Human Eye”, by BioRender.com (2022). Retrieved and edited from https://app.biorender.com/biorender-templates)

The outcomes of clinical trials testing for the safety and efficacy of neuroprotective agents demonstrate clear challenges in the aspect of drug delivery [146]. Poor drug delivery could be a factor largely contributing to the failure of the drug in clinical studies. The most common administration routes for glaucoma drug delivery are intravenous, subcutaneous, topical, and oral [155]. However, through these routes, ocular drugs are prone to absorption into the systemic circulation, which may result in low dose delivery to the target tissue and increase systemic risks. The low dose delivery could be also due to the poor solubility of the drug entailing high degradation rate and the failure to pass through the cornea and across the blood-retina barrier [156]. For topical administration, the corneal and the non-corneal routes control and influence the absorption [22]. The course that the drug molecules take towards the target cell in the intraocular environment starts with their passive diffusion via barriers formed by tight interconnected junctions. The components of these barriers include the precorneal pocket, corneal epithelium, the blood aqueous barrier, the retinal pigment epithelium and the blood capillary endothelial cells (choroidal barrier), all of which inevitably restrict the permeation of drug molecules into the intraocular chamber where they are to carry out their pharmacological action, resulting in an inefficient therapy [157]. Even if it were perfectly efficient, this trajectory would hardly allow for the access of the drug to the posterior area of the eye, where RGCs and the optic nerve reside. The most common route of administration to treat this posterior segment of the eye in experimental studies has been through intravitreal injection [158]; this is an immediate and direct route, which can increase the therapeutic drug delivery to the vitreous cavity and bypass the aforementioned barriers [159]. The intravitreal route is safe and effective, but due the invasiveness of the procedure, it is accompanied by side effects such as elevated IOP, cataract formation, bleeding and the risk of ocular infections [159]. This route is particularly problematic because drugs can be prevented from reaching the target tissue by the posterior vitreoretinal interface, including the inner limiting membrane of the retina and the vitreous cortex [160]. Figure 3 shows a basic schematic of the eye with anatomical barriers and common routes of drug delivery.

The eyes are easily accessible in terms of delivering the drug into the body, yet the drug distribution is one of the most challenging endeavors. Therefore, the development of safer and more efficient drug delivery systems is vital for therapeutic purposes. Research conducted to date address these challenges and there is a growing consensus that the characteristics of ideal drug carriers are as follows [161, 162]:

- Particle size reduction and direct interaction with target cells or tissues (adhesive properties);
- Improved drug retention time in the precorneal area and promoted drug tissue permeation as well as optimal tissue absorption;
- Improved solubility of poorly soluble drugs (e.g., oral delivery of lipophilic drugs into the systemic circulation) and prolonged drug shelf-life;
- Biodegradability and biocompatibility;
- Absence of irritant features to reduce the drug dosing regimens and improve patient adherence to medication;
- Protection of sensitive therapeutic molecules (e.g., small molecule drugs and bioactive agents) against degradation agents such as enzymes;
- Targeted and controlled drug release characteristics that provide dose accuracy, reducing or preventing side effects and being ideal for long-term treatments.

The above criteria could be achieved by implementing NPs as drug carriers in ophthalmic drug formulations. This platform transports the drug molecules across biological barriers by physically or chemically attaching the molecules to the NPs [163]. The large surface-to-volume ratio of NPs, along with other chemical characteristics, enables mucoadhesive properties that aid in drug adhesion to the mucosa of the corneal tissue, hence potentially increasing the drug contact time with the ocular tissue [164]. Apart from the abovementioned, NPs have been shown to improve patient self-care and compliance in terms of reducing the frequency of topical eye drop instillations, ultimately reducing the required doses and the risk of adverse effects [161]. NPs for ocular drug delivery can not only improve the solubility of drugs so as to reach the posterior segment of the eye, but also enhance the cellular uptake and protect the drug from degradation [165]. By formulating NPs with currently available ophthalmic solutions or investigational drugs, a greater potential for an effective glaucoma therapy can be ascertained in the future. Tabular overview of current investigated polymeric and lipid based-NPs with incorporated ophthalmic substances as compared to pure substances in ocular tissues is presented in Table 2.

**Table 2:** Polymeric- and lipid-based conjugated NPs as carriers of ophthalmic substances

Nanoparticle’s formulation	Substances	Size of NPs (nm)	Surface charge (mV)	Route of delivery	Platform	Advantages	Refs.
Polymeric-based NPs							
PLGA	Sparfloxacin	181 to 232	+ 22	Topical instillation (nanosuspension)	In vitro In vivo	Reduced IOP, improved precorneal residence time, enhanced ocular penetration, and good eye tolerance	[231]
PLGA coated-chitosan gel	Sparfloxacin	181	NR	Topical instillation (laden in situ gel)	In vivo	Reduced IOP, improved drug penetration, promoted sustained drug release, and prolonged drug retention time	[232]
PLA coated-PEG	Acyclovir	51.2 to 131.5	− 14.7	Topical instillation (conjunctival sac)	In vitro In vivo	Reduced IOP, prolonged retention time, and improved drug efficacy	[233]
Poly(amidoamine) (PAMAM) coated-PLGA	Brimonidine tartrate; timolol maleate	258	− 28.8	Topical instillation	In vitro In vivo	Reduced IOP (≥ 18%), non-toxic, prolonged time, increased drug bioavailability, controlled and slow release (~ 5 weeks)	[234]
PLGA-vitamin E-tocopheryl polyethylene glycol 1000 succinate	Brimonidine tartrate	115.72 ± 4.18	− 11.80 ± 2.24	Topical instillation (in situ gel)	In vitro Ex vivo In vivo	Reduced IOP (~ 8 h), improved precorneal residence time, non-irritant, and sustained corneal release	[235]
PLGA-phosphatidylserine (PSA) (core shell NP)	Brinzolamide	571.00 ± 27.02	− 27.45 ± 2.98	Subconjunctival injection	In vitro Ex vivo In vivo	Reduced IOP, enhanced coronial drug penetration, sustained release, high encapsulation efficiency, and non-toxic	[175]
Chitosan	Brimonidine tartrate	270 to 370	+ 26.2 to + 29.8	Topical instillation	In vitro In vivo	Reduced IOP, non-irritant and safe, provided mucoadhesive effect, prolonged retention time, and sustained drug release	[236]
Chitosan coated-carbopol	Pilocarpine	294	+ 55.78	Topical instillation	In vitro In vivo	Prolonged drug release with high bioavailability (unloaded > 90% drug in ~ 4 h)	[237]
Chitosan coated-PLA	Rapamycin	300	+ 30.3	Topical instillation	In vitro In vivo	High precorneal retention time (50% within 12 h), prolonged drug release, and significant immunosuppressive effects	[238]
Chitosan coated-PLGA	Triamcinolone acetonide	334.00 ± 67.95 to 386.00 ± 15.14	+ 26 to + 33	Topical instillation	In vitro	High drug encapsulation (55–57%) and controlled drug released (27 h)	[185]
Chitosan coated-sodium alginate	Gatifloxacin	205 to 572	+ 17.6 to + 47.8	NR	In vitro	Rapid drug release in the first hour but prolonged release over 24 h	[239]
Chitosan coated-cyclodextrin	Econazole nitrate	90 to 673	+ 22 to + 33	Conjunctival sac (instillation)	In vitro In vivo	Prolonged drug release (~ 50% within 8 h) and high bioavailability	[240]
Chitosan-coated sodium alginate/chitosan	5-Fluorouracil	329 to 505	+ 18.5 to + 28.9	Topical instillation	In vitro In vivo	Increased drug bioavailability and prolonged release (~ 8 h)	[241]
Lecithin coated-chitosan	Natamycin	213	+ 43	Conjunctival sac (instillation)	In vitro In vivo	Increased drug retention time (> 64% released over ~ 7 h), reduced clearance, improved mucoadhesive properties, and fewer doses required	[242]
HA-modified chitosan	Timolol maleate; dorzolamide hydrochloride	118.4 to 143.9	+ 29.0 ± 8.7	Topical instillation	In vitro Ex vivo In vivo	Reduced IOP, improved mucoadhesive properties (~ 91%), provided controlled drug delivery, slow but sustained release, and non-irritant	[243]
Poly(γ-glutamic acid) (γ-PGA)-l-phenylalanine (-Phe)	Dexamethasone	200	− 25	Topical instillation	In vitro In vivo	Efficient drug uptake by cultured macrophages/microglia and inhibited microglia at 24 h post-treatment	[244]
Ethylcellulose	Melatonin	147.4 to 179.6	− 25 to − 30	Topical instillation	In vivo	Greater cornea penetration and RGC survival at 9 days post-treatment	[245]
Eudragit	Brimonidine tartrate	143.9 to 702.2	NR	Topical instillation	In vitro Ex vivo In vivo	Reduced IOP (~ 2 to 3 h longer than 1 h of commercialized eye drop) and prolonged drug release	[246]
Lipid-based NPs							
SLNs	Tobramycin	70 to 80	NR	Topical instillation (lower conjunctival sac)	In vivo	Prolonged drug release and retention (~ 4 h) and high bioavailability	[247]
	Baicalin	91.42 ± 1.02	− 33.50 ± 1.28	Topical instillation	In vitro In vivo	Prolonged drug release (~ 62% after 3 h and the remaining gradually within 10 h) and high corneal permeability	[248]
SLNs-coated modified Chitosan	Methazolamide	143.9 to 702.2	+ 31.3 ± 1.7	Topical instillation	In vitro Ex vivo In vivo	High ocular penetration, sustained drug release (~ 8 h), fewer doses required, and enhanced patients’ adherence	[249]
SLNs modified phospholipids	Timolol maleate	37.7 to 47.2	NR	NR	In vitro Human cornea construct	Enhanced drug bioavailability and encapsulation rate (> 44%)	[250]
SLN-PEGylated	Latanoprost	105 to 132	− 29.1 to − 26.7	Topical (contact lens)	In vitro In vivo	High drug uptake, sustained drug release, and safe	[251]
	Travoprost	221 to 257	− 27.3 to − 20.4	Topical (contact lens)	In vitro In vivo	High drug uptake, sustained release (> 144 h), safe and non-irritant	[178]
SLN-coated Poloxamer 188 and glycerol monostearate (solid lipid)	Chloramphenicol	248	− 8.74	NR	In vitro	Increased encapsulation efficacy (> 83%) controlled and prolonged drug release (> 48 h)	[252]
SLN-coated glycerol monostearate	Bimatoprost	148.4 ± 1.25	− 20.8 to − 14.1	Topical instillation (in situ gel)	In vitro Ex vivo In vivo	Prolonged drug release, increase in precorneal residence time, non-irritant, safe with low corneal toxicity, and stable (> 1 month)	[253]
SLNs-coated Compritol 888	Indomethacin	140 ± 5	+ 21.0 ± 1.8	NR	In vitro	Increased drug stability, encapsulation (72%), and corneal permeability; stable (> 1 month)	[254]
NLCs	Mangiferin	51.39	− 36.5 ± 1.5	Probe implantation	In vitro In vivo	Prolonged drug release (~ 3 months), increased corneal permeability and pericorneal retention time, high encapsulation efficacy (> 88%), and bioavailability	[255]
	Brimonidine	100 to 140	− 31.1 to − 33.7	Topical (contact lens)	In vitro In vivo	High drug uptake, sustained release (> 144 h), and safe	[177]
NLCs coated-Lutrol F 68 (surfactant), squalene (lipid) and Precirol ATO 5 (lipid)	Triamcinolone	198.73	− 29.30 to − 45.60	Conjunctival sac (instillation)	In vivo	No signs of ocular toxicity and improved encapsulation efficacy (94.82 ± 1.12%)	[256]
NLCs-coated Miglyol 812, castor oil, and stearic acid (lipid)	Flurbiprofen	228.3	− 33.3	Topical instillation	In vitro In vivo	Prolonged drug release, high encapsulation efficacy (~ 90%) and minimal irritation	[257]
NLCs-coated Chitosan, with ethanol (co-surfactant), Tween 80 (surfactant), oleic acid (liquid lipid), and Compritol HD 5 ATO (solid lipid)	Ofloxacin	244	− 4.630 ± 0.259	Topical (Ocular inserts in *cul de sac*)	In vitro, microbiological test Ex vivo In vivo	Enhanced precorneal permeation, retention time (~ 24 h) and enhanced drug efficacy, and reduced frequency application	[258]
Lipid NPs coated-phospholipids	Diclofenac sodium	276	− 12 to − 42.6	NR	In vitro Human cornea construct	Increased drug encapsulation (~ 94%), corneal penetration, and prolonged drug release	[259]
Lipoamino acid-modified NPs	Connexin43 mimetic peptide	NR	NR	Intravitreal injection	In vivo	Enhanced neuroprotection after retinal ischemia	[260]

*ATO* = atomic grade; *IOP* = intraocular pressure; *NPs* = nanoparticles; *NR* = not reported; *PEG* = poly(ethylene glycol); *PLA* = poly(lactic) acid; *PLGA* = poly lactic-co-glycolic acid; *NLCs* = nanostructured lipid carriers; *SLN* = solid lipid NPs

### Nanoparticles as ocular drug delivery systems

NPs are ultrafine solid structures that vary in morphology and have at least one spatial dimension in the range between 1 and 100 nm, and sometimes up to 500 nm, for larger particles. In the field of drug delivery, NPs are formulated to enhance the penetration and drug targeting of the active compound, while promoting a sustained release [166]. Due to their miniscule nature, NPs can often easily infiltrate the anatomical barriers in the CNS [164], such as the blood-brain and blood-retinal barriers, and thus directly provide a maximal drug bioavailability to the target cells [157]. In NPs, the drug-loading capacity is dependent on a few factors, including chemistry and microstructure, but also size, especially for NPs carrying their drug payload on the surface. Smaller NPs in these cases provide a higher loading capacity than the larger ones due to their higher specific surface area. NPs can exhibit a wide variety of morphologies, which help to serve the specific purposes to provide an effective therapy.

Multiple studies have attempted to develop drug-encapsulated NPs for the delivery to anterior and posterior segments of the eye. Conjugating ocular drugs onto NPs has been shown to boost eye permeation, particularly pass through the precorneal barrier [167]. In neurodegenerative diseases associated with inflammation, extensive studies have exploited drug-encapsulating NPs [168]. Some of the NPs that have been employed in neurodegenerative experimental studies, including polymeric and lipid based ones, have emerged as the key players in the domain of anti-inflammatory drug carriers [169].

**Fig. 4:**
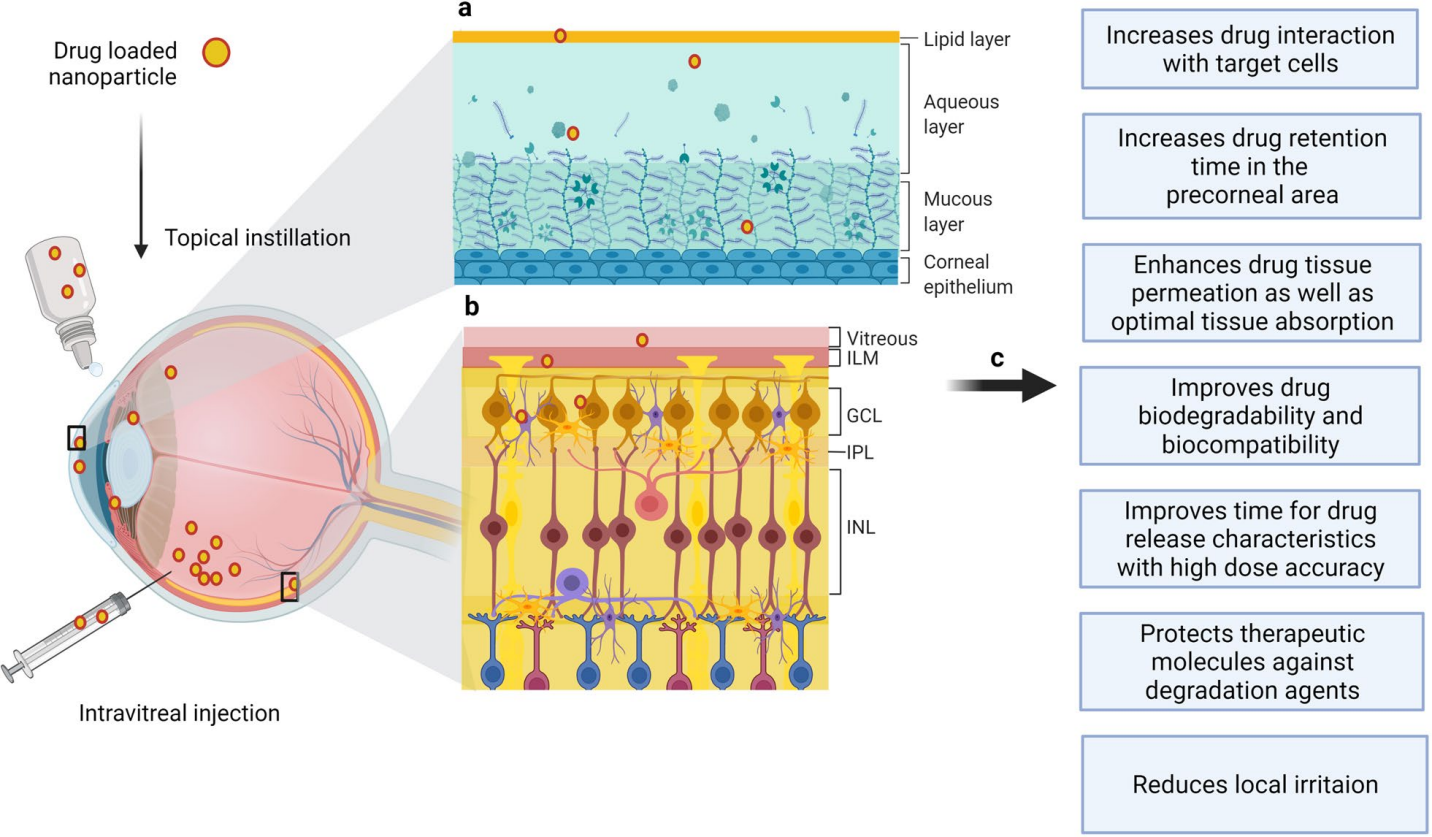
Overview of nanoparticle-mediated ocular drug delivery through the corneal barrier (**a**) and blood-retinal barrier (**b**). The potential advantages of the use of nanoparticles as an approach for improving current glaucoma medication (**c**) (Adapted from “Anatomy of the Human Eye”, “Structure of the Retina” and “Tear Film Structure”, by BioRender.com (2022). Retrieved and edited from https://app.biorender.com/biorender-templates)

Generally, in the drug delivery field for neurodegenerative and ocular diseases, NPs are most commonly made of soft carbonaceous materials, such as polymers and/or lipids [170]. Both lipid and polymeric NPs have successfully delivered drugs for several therapeutic purposes, while protecting the encapsulated drugs from enzymatic degradation and controlling their release. NPs made of natural or synthetic polymers and proteins [e.g., chitosan, poly(ethylene glycol) (PEG), polycaprolactone, sodium alginate, and albumin] usually take the form of finely dispersed latexes [171]. Compared to other nanomaterials such as the inorganic ones (e.g., zinc oxide or aluminum oxide), the former have caused a minimal eye irritation and prolonged retention of drugs, and thus allow for the circumvention of multiple medications and dose reduction [162]. However, compared to nanomicelles, a type of nanocarrier, polymeric-based NPs have been unable to escape the rapid loss of the instilled solution from the precorneal integument and the nasolacrimal drainage system. To overcome this limitation, NPs with mucoadhesive properties (i.e., chitosan and hyaluronic acid) were developed [172]. Of note, both polymeric- and lipid-based NPs have successfully delivered drugs for a number of therapeutic purposes, while protecting the encapsulated drugs from enzymatic degradation and controlling their release [166]. Figure 4 shows the benefits of drug loaded NPs administered through the corneal and blood-retinal barriers.

In the beginning of the application of NPs in ocular drug delivery, different carbon-based NPs were developed with the aim of producing a sustained drug release in the precorneal pocket. This was due to the majority of ophthalmic formulations being administered as eye drops, which in their conventional forms are poorly bioavailable on the corneal surface and intraocular tissues. Among the earliest NPs were those made of acrylic polymers such as poly-alkylcyanoacrylate (PACA), which extended the time of drug contact with the eye surface. There was increased drug action duration, however, this resulted in ocular toxicity. Later, polyacrylamide NPs began to replace PACA for the same purpose [173].

Polyester NPs (e.g., polycaprolactone) have emerged in the recent times as a key biodegradable material for ocular drug delivery, largely thanks to the acute tolerance of the ocular surface to them. At the same time, polyester NPs can increase drug efficacy. For example, an ophthalmic betaxolol, a beta-adrenergic blocking agent, displayed its optimal pharmacological effect when encapsulated by polycaprolactone (hydrophobic NPs) owing to its gradual release. NPs made of biodegradable materials such as hyaluronic acid or composed of hydrophilic polysaccharides (components of the vitreous body) have also been proven safe for incorporation into ophthalmic solutions. Other NPs composed of polymeric-based materials have also improved the drug delivery interaction with the cornea, and thus allowed for the controlled drug release and the treatment of the ocular disease of the outer segment. It was postulated that nanocarriers coated with bioadhesive polymers (e.g., PACA and cyclosporine-A) can enhance the penetration of the embedded drug and improve the stability in the lacrimal fluid, which has been shown to prevent the enzymatic degradation of the delivered drug [173].

Recently, several studies have been performed to upgrade these standard NP formulations by reforming NP surface properties such as coating with functional groups [155]. One example is lectin, a glycoprotein that exhibits extremely high binding affinities for specific carbohydrate groups present on the surface of corneal epithelial cells [174]. Accordingly, better tissue penetration was demonstrated for positively charged NPs, unlike in the case of negatively charged NPs, which get electrostatically repelled from the cell membrane. To improve the adhesion on the mucosal surface e.g., at the periocular and oral mucosa, for a sustained drug release and efficient absorption, NPs were formulated with different bioadhesive polymers [173].

Due to their optimal size for the penetration of ocular barriers, NPs usually do not impose eye irritation, thereby limiting the frequency of drug administration as well as maintaining sustained drug release [161]. Lately, there has been an increased emergence of reports on NPs (e.g., polymeric [175, 176] and lipid-based [177, 178]) for drug-eluting contact lenses and corneal implants. Such commercialized medical devices provide a sustained and burst drug release with high bioavailability to the anterior and posterior segments of the eye, which may improve patient adherence as compared to eye drop medications [179]. Furthermore, contact lenses are typically used to correct refractive errors (e.g., myopia and hyperopia), which may positively impact patients' adherence towards their treatment regiment, particularly for those with both errors and glaucoma. Despite potential benefits, this approach is associated with potential safety risks and other limiting factors pertaining to production and storage [179]. As a result, topical eye drops continue to be the preferred first-line treatment option for glaucoma. Nonetheless, a substantial increase of studies is now being conducted to address the drawbacks of drug-eluting contact lens, making it possible for delivering medications to the eye and commercialization in the future. Among many others, Chauhan et al*.* have been working extensively on developing novel loaded contact lenses employing diverse NPs formulations. While this is an intriguing topic, it is beyond the focus of this review. For further information on this subject, refer to the cited reviews by Chauhan and others [180–183] as well as their recent published works on the fabrication of ophthalmic drug-eluting contact lenses using various nanomaterials [180–182, 184–188].

In the posterior eye segment, prolonged drug delivery could be achieved with the application of NPs, depending on their size and characteristics of the surface. In addition, prolonged and effective transscleral drug delivery through intravenous administration (blood and lymph circulation) could be achieved by conjugating the right moieties onto NPs. Intravitreal administration, for example, enables macromolecular drugs to reach the retina and reduces systemic toxicities. Through this technique, the encapsulated drug molecules can be accumulated at the retinal pigment epithelium layer, and thus maximize the therapeutic effects [158]. Dexamethasone-loaded poly lactic-co-glycolic acid (PLGA) NPs administered intravitreally in rabbits, for example, elevated the cellular uptake with stable bioavailability in the vitreous fluid, chorioretina, and plasma compared to the unconjugated dexamethasone [189]. The same features were observed for human serum albumin NPs, where the conjugated drug molecules successfully infiltrated the retina layers through specific pathway in the Müller cells, relying on endocytosis and exocytosis [190]. Of note, more investigations are needed to ascertain the ocular tissue penetration of NPs loaded with high molecular weight drugs, especially through the vitreoretinal interface, which is one of the major obstacles to reach the inner retina upon intravitreal administration. Indeed, not all NPs allow the drug molecules to be efficiently dispersed through the vitreous fluid or pass through the inner limiting membrane. The efficiency of the therapeutics to cross this membrane are highly dependent on their ability to migrate from the injection site towards the retina, which is often determined by the size of the NPs along with their other physicochemical properties. For further reading on this subject, the reader is referred to the recent and ongoing work by Peynshaert et al. [50–52]. Nevertheless, in view of all that has been mentioned so far, one may suppose that NPs have the potential to serve as an effective intravitreal drug delivery system.

A few studies have been dedicated to the attempts to limit the drug clearance, given that most administered NPs pool in the liver and spleen and are removed by the reticuloendothelial system after the administration [191]. Interestingly, NPs are flexible and can escape opsonization by the macrophages if coated with extra surface layers, such as PEG [192]. By controlling properties of NPs, it is possible to achieve maximal therapeutic effects, minimal side effects as well as highest solubility for targeted drugs [192]. In order to prolong the drug retention and enhance its corneal permeability and bioavailability, it is important to select appropriate NPs in terms of their chemistry, size, shape, surface charge and other physicochemical properties [193]. Herein, the suitable biocompatible ocular drug delivery system depends on the target tissue, the route of administration, and the characteristics of the drug to be incorporated into the NPs. Despite all of the advantages, however, the high permeability of NPs pose a high risk, as shown in several brain studies [194]. For instance, zinc oxide NPs and the anatase phase of titanium dioxide can easily bypass the blood-brain barrier via multiple routes and induce neuroinflammation with the potential to be neurotoxic [195]. Hence, the careful selection of carriers is of prime importance when designing the drug delivery system utilizing NPs.

## Application of different nanoparticles as anti-inflammatory drug carriers

### Polymer-based NPs

**Fig. 5:**
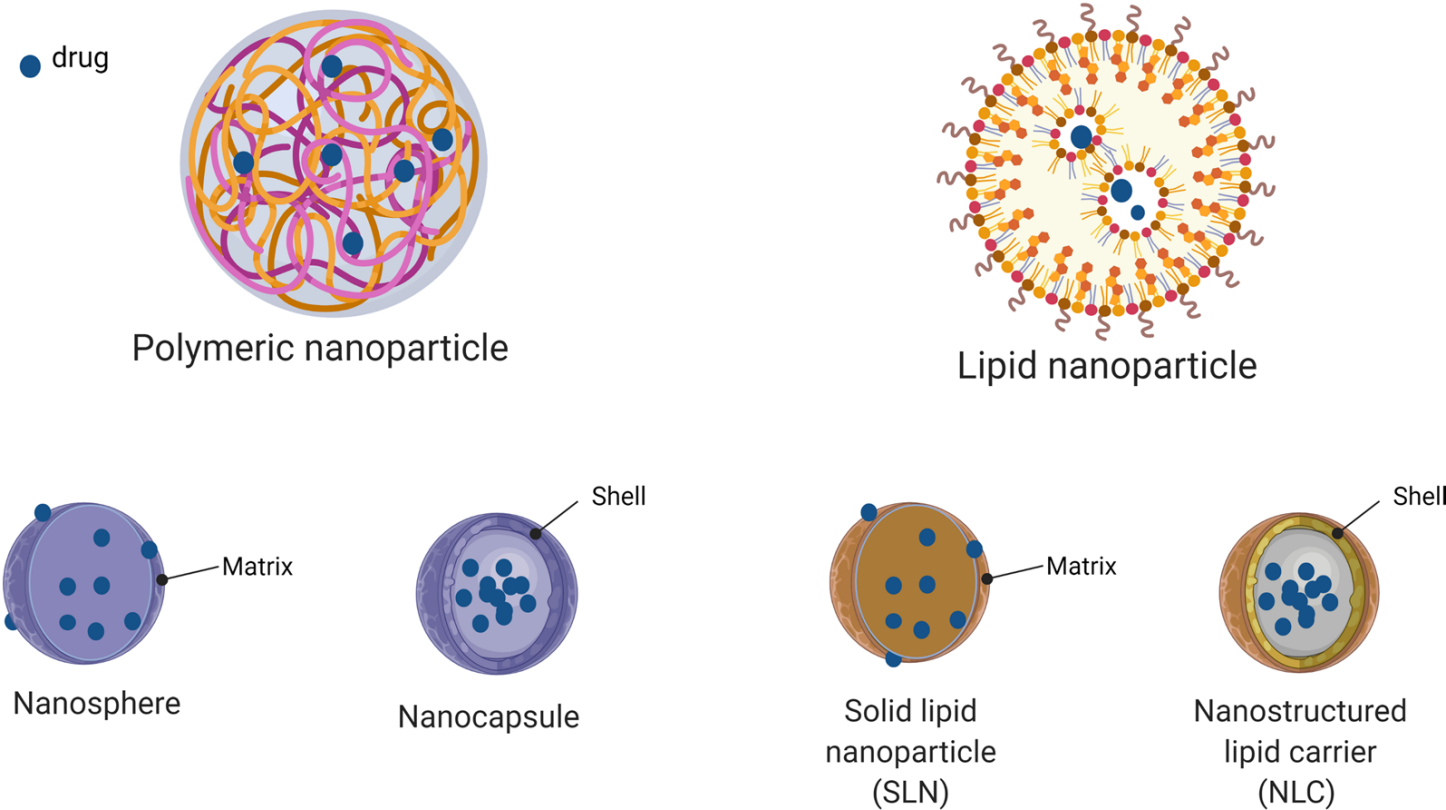
General view of drug-loaded polymeric (nanosphere and nanocapsule) and lipid (solid lipid nanoparticle and nanostructured lipid carrier) nanoparticles. Created with BioRender.com (2022)

Polymeric NPs have played a major role in the advancement of NP-mediated drug delivery, given that they have proven successful for alleviating numerous diseases [155]. Polymeric NPs can form nanocapsules (surface-vesicular systems) or nanospheres (matrix systems) depending on their internal structure and preparation method (Fig. 5). While the former systems contain a drug encapsulated within a liquid core cavity, the latter ones contain a structural polymeric matrix where the drug is physically and uniformly dispersed [196]. In addition to being incorporated inside a polymeric matrix, the drugs can also be adsorbed on the NP surface. These biodegradable NPs ranging from 10 to 100 nm are the most commonly studied in the ocular drug delivery field [173]. Polymeric NPs have been proven superior compared to other types of NPs for ocular application primarily due to their properties including biodegradability, lesser toxicity, similarity in stiffness compared to the soft tissues, good encapsulation capacity as well as controlled release manner, alongside biocompatibility and mucoadhesiveness [170]. Different types of polymeric NPs can be produced by directly processing different monomers or by using derived polymers obtained through polymerization [173]. They are also applicable in producing many different NPs, which can improve drawbacks of conventional drug delivery systems.

Polymeric NPs have been shown to improve the stability of easily volatile substances and may act as preservatives in ophthalmic solutions [197]. This results in a greater efficiency and effectiveness in transporting maximal concentrations of active pharmaceutical ingredients to the targeted site, making them an ideal choice for modifying drugs in cancer therapy. Apart from drugs, polymeric NPs have been used as gene delivery carriers [173]. Furthermore, high bioavailability of polymeric NPs is related to their ability to concentrate at a targeted spot via passive or ligand-mediated mechanisms. This method offers the possibility for reduction of required dose(s) and the side effects associated with them. Despite all these advantages, these types of NPs may still potentially cause toxicity due to the organic solvents incorporated in the final formulation and deterioration of the polymer, which may produce systemic pernicious aftermath effects [171]. Nevertheless, most polymeric NPs provide beneficial properties required for the use in drug delivery in ophthalmology, fundamentally relying on the capability to retain drugs in ocular tissues. By interacting with mucin, mucoadhesive polymers help to minimize the elimination of drugs from the surface of the eye and therefore can increase the drug bioavailability at the precorneal area. Several examples of these types of polymers with modified surface characteristics are PLGA, PEG, poloxamers, poloxamines, hyaluronic acid, chitosan, sodium alginate and polyacrylic acid [170].

One of the widely utilized materials for polymeric NPs is PLGA [198]. PLGA has been approved by the United States Food and Drug Administration (US FDA), owing to its high biodegradability and biocompatibility demonstrated by long-term clinical trials [199]. In in vitro studies, dexamethasone-encapsulated PLGA has been shown to successfully bypass the human placenta with high bioavailability [200]. An empirical study conducted using prednisolone on C6 cells, a type of cancer cell resembling astrocytes, has shown that prednisolone-encapsulated PLGA NPs attenuated pro-inflammatory cytokines including TNF-α and nitric oxide, surpassing the effects of naked prednisolone [201]. On the other hand, in a glaucoma study using the rabbit’s cornea, a combination of dexamethasone and melatonin loaded PLGA NPs has been observed to significantly reduce the level of IOP [189]. This study associated the neuroprotective effect with the enhanced corneal penetration and sustained release of dexamethasone and melatonin by NPs. Interestingly, PLGA NPs are capable of encapsulating several active pharmacologic drugs simultaneously such as dexamethasone and melatonin for further improvement in delivering the drug [202]. For example, in an ex vivo rat brain tissue study, PLGA NPs coated with PEG exhibited rapid infiltration compared with uncoated NPs, indeed suggesting that coated NPs improved drug permeation [202].

In addition, prolonged delivery of therapeutic drugs is one of the most significant factors for successful neuroprotective therapy in glaucoma. To reach its target, NPs should be able to avoid the uptake by the mononuclear phagocytic system (MPS) of the host [203], which is responsible for opsonization and phagocytosis. Upon delivering the drugs, NPs are often specially designed to avoid or harness the MPS to reduce inflammatory effects, subsequently improving the payload delivery and the drug therapeutic efficacy [203]. It is established that PEG-coated PLGA NPs improve drug uptake and clearance. For instance, hydrophobic PLGA NPs coated with hydrophilic PEG exhibited an antagonism against opsonization and phagocytosis along with prolonged circulation time in the blood compared with NPs prepared without PEG [204]. Moreover, in a study done using anthocyanin, a phenolic compound with a high antioxidant activity, PLGA-PEG NPs encapsulating this compound were shown to effectively abolish the expression levels of inflammatory markers including NF-kB, TNF-α, and inducible nitric oxide synthase (iNOS) as well as apoptotic markers such as bcl-2 associated x (Bax), b-cell lymphoma-2 (Bcl-2), and caspase-3 protein against amyloid beta peptide 1-42 (Aβ1-42)-induced neurodegenerative effects in SH-SY5Y cell lines [205]. Taken together, these PEG-coated PLGA NPs can improve therapeutic drug delivery by inhibiting both neuroinflammatory and neuroapoptotic pathways. Although the delivery of anti-inflammatory drugs with PLGA has yet to be studied in glaucoma, this highlights its strong potential as a nanocarrier, particularly for the treatment of neuroinflammation.

### Lipid-based NPs

Lipid-based nanocarriers are at the forefront of the rapidly developing drug delivery systems for various diseases. Here, we hypothesize that the lipid-based NP system is also one of the most promising drug delivery systems for treating glaucoma, improving drawbacks associated with the conventional treatment. Topical liposomal nanocarrier is one widely used lipid-based nanocarrier in preclinical and early clinical studies, efficiently delivering ophthalmic solutions such as timolol maleate into the vitreous and retina [206]. In many respects, lipid NPs are superior carriers compared to liposomes and polymeric NPs. The main benefits of these NPs are that they do not require organic solvents, which generate toxic degradation products, to be formulated unlike the polymeric NPs. As a result, they exhibit low in vivo toxicity as well as protect and stabilize the loaded drug molecules from degradation, while offer the controlled drug release capacity.

Lipid NPs are composed of o/w (oil-in-water) emulsions, which is a combination of a lipid nucleus with an amphiphilic surfactant acting as the stabilizer. As such, they are able to transmit both hydrophilic or hydrophobic drugs [207]. Lipids in a liquid state can transform to a solid state of various structures (e.g., steroids, monoglycerides, diglycerides, and triglycerides) and be dispersed in an aqueous solution at room and body temperature. In this context, lipid NPs are the aqueous dispersion of spherical vesicles made of ionized lipids with a positive charge at the neutral pH. They range in size from 40 to 1000 nm and can be classified into two categories: solid lipid NPs (SLN) and nanostructured lipid carriers (NLC) (Fig. 5) [208].

Initially, SLNs were formulated to improve the available drug delivery systems such as polymeric NPs and liposomes. SLNs are made of solid lipids derived from water, co-emulsifiers, and emulsifiers. As the more improvised version of drug carriers than the aforementioned ones, SLNs deliver several advantages, including the improved drug loading capacity, prolonged duration of drug release, higher drug bioavailability with a better stability for unstable molecules against chemical degradation, improved safety as well as good cost-effectiveness ratio, particularly for high-scale production [207]. SLNs also enhance the corneal absorption and conjunctival uptake, as shown in studies done on anterior and posterior eye tissues, and thus extend the drug retention period [209–211]. These studies have shown the potential of implementing SLNs formulation in clinical practice.

Nanostructured lipid carriers (NLCs), on the other hand, were designed as the alternatives to compensate for the prominent drawbacks of SLNs, such as their very limited drug-loading capacity [212]. NLCs are formed from a mixture of solid and liquid lipids that adopts an amorphous solid matrix state at room and body temperature. NLCs exhibit high drug tolerance due to the physiological and biodegradable lipids constituting them. Moreover, NLCs offer a higher drug loading capacity and extended drug release time compared to the SLNs, which includes both hydrophilic and lipophilic drugs [207]. In general, there are three types of NLCs: the imperfect, non-shaped (amorphous) and the multiple structures. The imperfect type refers to a mixture of fatty acids blended to create several lipid formations in a crystal structure (disorganized matrix) with gaps which provide the space for lipophilic drugs to enter the particles. On the contrary, the amorphous type does not have a crystalline matrix, hence it prevents premature drug ejection. Lastly, the multiple structures type consist of several compartments of a liquid lipid in a matrix of a solid lipid. This NLC type is utilized to avoid drug decomposition caused by the solid lipid. The development of NLC formulations has been demonstrated in ocular drug delivery to both the posterior and anterior parts of the eye [213]. For example, Luo and co-authors reported NLC chitosan-coated genistein formulation delivered via a topical administration, which enhanced the transcorneal penetration with an increased bioavailability of the drug molecules in the aqueous humor compared to the conventional solution [214]. Furthermore, triamcinolone acetonide encapsulated NLC showed enhanced therapeutic efficacy in mice. The developed formulation was able to reach the posterior segment of the eye via the corneal and non-corneal pathways upon topical administration [215]. These studies prove that the NLC formulations are good candidates for ocular drug delivery for treating glaucoma.

Overall, in comparison with other particulate systems, lipid NPs provide many advantages. From the commercial perspective, lipid NPs are feasible and easy to engage in a large-scale production [216]. Since lipids are biocompatible, lipid NPs are highly tolerated by the body. Furthermore, drug formulations with emulsifiers could have a better stability profile for both hydrophilic and lipophilic drugs, meaning they would be able to control and extend their retention time in the body. Besides, the important characteristics of lipid NPs including the optimal particle size, surface charge, drug entrapment efficiency, drug encapsulation and elimination, which enable them to protect the incorporated drugs from enzymatic degradation in the eye. This eventually provides a good adhesion onto the cornea/periocular tissues. Since they are composed of lipids, these NPs help to reach the lipid layer of the tear film, which directly improves the drug delivery and drug bioavailability for topical instillation. The natural affinity of lipid NPs for the lipid layers can be further augmented by endowing the given fatty acid chains with a positive surface charge, given that cationic lipids and surfactants extend the retention time of emulsion drops on the epithelial layer of the cornea [207]. Interestingly, studies done on the cytotoxicity of both SLNs and NLCs demonstrate that they are well tolerated and do not cause irritation to the ocular tissue. Still, non-ionic surfactants are occasionally required to minimize the toxic effects of the drug conjugated to lipid NPs [212]. Yet, these NPs can facilitate the passage of non-ionized drugs across various barriers, such as the precorneal film, while maintaining the neutral form of the encapsulated drug in its active form [212].

## Conclusion

Several biological and nanoparticle-assisted agents have been evaluated in the experimental models of glaucoma, but none of them have passed the clinical trials. This possibly has to do with the complex molecular processes governing neuroprotection that are yet to be elucidated. Turbulence in the immune response surveillance is regarded as the prime source of the disease progression, including that in autoimmune and other neurodegenerative diseases. We, therefore, have discussed the prospect of tackling the inflammatory response at the early stages of glaucoma. Traditionally, the absence of the symptoms rarely prompts a clinical evaluation, let alone a treatment for the disease, notwithstanding the fact that most forms of glaucoma are asymptomatic in the early stages. It is also conceivable that IOP-lowering treatments may not be effective in circumventing the progression of the disease, as they lead to the progressive damage to the RGCs. The ineffectiveness of the available treatments has contributed to the improvement and development of several drug delivery systems. In ocular drug delivery systems, NPs have a vast applicability and potential to improve the efficacy of the current available treatments for glaucoma. NPs may enhance the current therapies by modulating drug solubility and subsequently enhancing bioavailability. They may also assist the drugs to permeate the critical barriers *en route* to their ocular target, but also extend the drug delivery timescale. The adverse effects at large may be minimized too as targeted delivery and improved bioavailability reduce the need for higher doses. Therefore, combining NPs with biological or small-molecule agents with the ability to counteract the inflammatory response in glaucomatous neurodegeneration can potentially move the field of glaucoma therapy forward.

## Data Availability

Not applicable.

## Abbreviations

Aβ142: Amyloid beta peptide 1-42
AMD: Age-related macular degeneration
Ang II: Ang II angiotensin 1, 2
AP-1: Activator protein 1
AT1-R: Angiotensin type 1 receptor
Bax: Bcl-2 associated x
Bcl-2: B-cell lymphoma-2
BDNF: Brain-derived neurotrophic factor
C1, C3, C5, C1Q: Complement component 1, 3, 5, 1Q
CD3: Cluster of differentiation 3
CNS: Central nervous system
COX-2: Cyclooxygenase-2
EGCG: Epigallocatechin-gallate
ET-1: Endothelin-1
FasL: Fas ligand
GBE: Ginkgo biloba extract
GFAP: Glial fibrillary acidic protein
IL-1β, IL-6, IL-8, IL-18: Interleukin 1β, 6, 8, 18
iNOS: Inducible nitric oxide synthase
IOP: Intraocular pressure
IP10: Interferon gamma-induced protein 10
Jak: Janus kinase
MD: Mean deviation
MgAT: Magnesium acetyltaurate
MIP-1α, MIP-β, MIP-2: Macrophage inflammatory protein 1α, β, 2
MPS: Mononuclear phagocytic system
NF-κB: Nuclear factor kappa B
NLCs: Nanostructured lipid carriers
NLRP1, NLRP3: NOD-, LRR-family pyrin domain containing 1, 3
NMDA: *N*-Methyl-d-aspartate
NTG: Normal tension glaucoma
OHT: Ocular hypertension
ONH: Optic nerve head
PACA: Poly-alkylcyanoacrylate
PAMAM: Poly(amidoamine)
PEG: Poly(ethylene glycol)
PLGA: Poly lactic-co-glycolic acid
POAG: Primary open-angle glaucoma
NPs: Nanoparticles
RAAS: Renin-angiotensin aldosterone system
RGC: Retinal ganglion cell
ROS: Reactive oxygen species
RNFL: Retinal nerve fiber layer
RSV: Resveratrol
SIRT1: Sirtuin 1
SLNs: Solid lipid nanoparticles
SOD-2: Super oxide dismutase 2
TLR1, TLR4: Toll-like receptor 1, 4
TNF-α: Tumor necrosis factor-alpha
